# Prognostic significance of loss of heterozygosity at loci on chromosome 17p13.3-ter in sporadic breast cancer is evidence for a putative tumour suppressor gene

**DOI:** 10.1038/sj.bjc.6690427

**Published:** 1999-05

**Authors:** D S Liscia, R Morizio, T Venesio, C Palenzona, M Donadio, R Callahan

**Affiliations:** Servizio di Anatomia Patologica, Dipartimento Oncologico ASL-1, Torino, CAP 10123, Italy; Oncogenetic Section, LTIB, Bethesda, MD, USA

**Keywords:** breast cancer, chromosome 17p13.3, LOH, survival

## Abstract

Several studies indicate that the short arm of chromosome 17 is one of the most frequently altered regions in sporadic breast carcinomas (45–60%). In the present report the 17p13.3-ter locus in tumour DNA of breast cancer patients, along with their matching normal lymphocyte DNA, have been mapped with four markers (D17S5, D17S379, ABR and D17S34), spanning nearly 3 cM of the telomer. Sixty-five of 143 heterozygous tumours had lost at least one of the markers at the minimum region of loss (45%). High levels of loss of these distal markers on 17p13.3 are independent of TP53 mutations and are associated with tumour cell proliferation. A follow-up period of over 7 years demonstrates that loss of these markers correlates both with disease-free (*P* = 0.004) and overall survival (*P* = 0.007). In addition we show that for disease-free survival the prognostic power of this genetic alteration is second only to axillary lymph node involvement (3.1 vs 6.3 relative risk), and is a better predictor than the mutational status of TP53 (1.6 relative risk). Our results are further evidence of the presence, within the region, of at least a second tumour suppressor gene distal to TP53, that might be targeted by deletions. © 1999 Cancer Research Campaign


*British Journal of Cancer* (1999) **80**(5/6), 821–826
© 1999 Cancer Research Campaign
Article no. bjoc.1998.0427

### Prognostic significance of loss of heterozygosity at loci on chromosome 17p13.3-ter in sporadic breast cancer is evidence for a putative tumour suppressor gene

**DS Liscia[1], R Morizio[1], T Venesio[1], C Palenzona[1], M Donadio[1] and R Callahan[2]**

[1]Servizio di Anatomia Patologica, Dipartimento Oncologico ASL-1, via Cavour 31, Torino, CAP 10123, Italy; [2]Oncogenetic Section, LTIB, National Cancer Institute, Bethesda, MD, USA

**Summary** Several studies indicate that the short arm of chromosome 17 is one of the most frequently altered regions in sporadic breast carcinomas (45–60%). In the present report the 17p13.3-ter locus in tumour DNA of breast cancer patients, along with their matching normal lymphocyte DNA, have been mapped with four markers (D17S5, D17S379, ABR and D17S34), spanning nearly 3 cM of the telomer. Sixty-five of 143 heterozygous tumours had lost at least one of the markers at the minimum region of loss (45%). High levels of loss of these distal markers on 17p13.3 are independent of TP53 mutations and are associated with tumour cell proliferation. A follow-up period of over 7 years demonstrates that loss of these markers correlates both with disease-free ($P$ = 0.004) and overall survival ($P$ = 0.007). In addition we show that for disease-free survival the prognostic power of this genetic alteration is second only to axillary lymph node involvement (3.1 vs 6.3 relative risk), and is a better predictor than the mutational status of TP53 (1.6 relative risk). Our results are further evidence of the presence, within the region, of at least a second tumour suppressor gene distal to TP53, that might be targeted by deletions.

**Keywords:** breast cancer; chromosome 17p13.3; LOH; survival

Breast cancer development, as in other types of tumours, is thought to be the result of the unmasking of one or more recessive tumour suppressor genes by somatic alterations (Knudson, 1985). Three familial forms of breast cancer, caused by mutations of the BRCA1, BRCA2 and TP53 genes, are the prototypes for this kind of carcinogenesis. Among tumours from patients with inherited BRCA1 and BRCA2 mutations, over 90% have lost the wild-type alleles (Neuhausen and Marshall, 1994; Gudmundsson et al, 1995); however, both susceptibility genes are infrequent targets for somatic inactivation in sporadic breast cancer (Lancaster et al, 1996). TP53 is another gene that, within the Li–Fraumeni syndrome, also is responsible for an inherited predisposition to breast cancer (Sidransky et al, 1992). The p53 gene is mutated in about 22–27% of sporadic breast tumours (Sjögren et al, 1996). Several studies have been published in an effort to estimate the frequency of somatic loss of wild-type TP53 alleles, before it was understood that point mutated p53 could have a dominant-negative action (Milner and Medcalf, 1991) and therefore could modify cell functions even if one allele alone was affected. Most of these reports had initially used the highly informative YNZ22.1 (D17S5) marker, thought to map close to TP53, on the short arm of chromosome 17. It later became clear that the D17S5 locus is actually located on band 17p13.3, approximately 20 cM distal to the TP53 gene (Coles et al, 1990). At this locus, a high frequency of allelic losses (as high as 60%) has been detected in sporadic (Mackay et al, 1988) as well as in familial breast tumours (Lindblom et al, 1993). In breast carcinomas 17p13.3 loss of heterozygosity (LOH) is independent of TP53 point mutations and is associated with a high S phase index (Merlo et al, 1994). A previous report on a panel of primary tumours from stage I–IV breast cancer patients have shown than LOH of D17S5 is associated with disease-free survival (Nagai et al, 1994). The general hypothesis being tested in our study is that allele losses at a specific chromosome location, framed by a well-mapped set of markers, could be associated with the clinical course of breast cancer. Specifically, a positive association with 17p13.3-ter LOH would provide further evidence for the existence, in this locus, of a tumour suppressor gene whose mutation conferred a more aggressive phenotype.

*Received 11 November 1998*
*Received 27 November 1998*
*Accepted 9 December 1998*

*Correspondence to:* DS Liscia

### MATERIALS AND METHODS

### Patients and tumour samples

Between April 1987 and November 1991 160 primary breast carcinomas were removed surgically by either mastectomy or lumpectomy from untreated patients with stage I, II and III disease at the S Giovanni Vecchio Hospital, Torino, Italy. Following histological diagnosis made on frozen sections, tumour samples were dissected quickly to remove most of normal tissue and stored at –80°C. Peripheral blood was collected for all the patients whose tumour was genetically analysed. A total of 152 patients fulfilled the criteria for inclusion in this study; eight patients that had developed a second carcinoma in the contralateral breast were not considered eligible and were withdrawn. Thirty-nine patients received adjuvant chemotherapy (CT) according to the Milan protocol (Bonadonna et al, 1986). Most of the tumours were infiltrating ductal carcinomas ($n$ = 132), 16 tumours were infiltrating lobular carcinomas, 3 were mucinous and one a medullary carcinoma. The median follow-up duration time was 6.1 (range 0.7–8.6) years.

**Table 1** Association between LOH at proximal and distal markers of the 17p13.3 chromosome

| | D17S5/ D17S379 | | | | |
|---|---|---|---|---|---|
| | Normal | | LOH | | |
| D17S34/ABR | *n* | (%)[a] | *n* | (%)[a] | |
| Normal | 43 | (75.4) | 14 | (24.6) | $P$ = 0.00001[b] |
| LOH | 9 | (25.7) | 26 | (74.3) | |

[a]Row percentages. [b]By $\chi^2$ analysis.

**Table 2** Association between breast cancer clinicopathological variables and genetic alterations of chromosome 17p13.3

| | 17p13.3 Normal | | 17p13.3 LOH | | |
|---|---|---|---|---|---|
| Variable | *n* | (%)[a] | *n* | (%)[a] | *P*-value[b] |
| T (≤ 2 cm) | 36 | (63.2) | 21 | (36.8) | 0.1 |
| T (> 2 cm) | 42 | (48.8) | 44 | (51.2) | |
| N– | 34 | (52.3) | 31 | (47.7) | 0.5 |
| N+ | 44 | (58.7) | 31 | (41.3) | |
| Grade I | 4 | (80.0) | 1 | (20.0) | 0.1 |
| II | 40 | (56.3) | 31 | (43.7) | |
| III | 20 | (43.5) | 26 | (56.5) | |
| Stage I | 17 | (58.6) | 12 | (41.4) | 0.6 |
| II | 58 | (54.2) | 49 | (45.8) | |
| III | 3 | (75.0) | 1 | (25.0) | |
| Adjuvant CT– | 59 | (54.6) | 49 | (45.4) | 0.9 |
| Adjuvant CT+ | 19 | (54.3) | 16 | (45.7) | |
| ER– | 48 | (56.5) | 37 | (43.5) | 1.0 |
| ER+ | 27 | (55.1) | 22 | (44.9) | |
| BrdU | | | | | |
| Low | 45 | (65.2) | 24 | (34.8) | 0.02 |
| High | 33 | (45.2) | 40 | (54.8) | |
| Ki-67 | | | | | |
| Low | 34 | (56.7) | 26 | (43.3) | 0.8 |
| High | 41 | (53.2) | 36 | (46.8) | |
| TP53 | | | | | |
| Wild-type | 36 | (55.4) | 29 | (44.6) | 0.3 |
| Mutated | 10 | (41.7) | 14 | (58.3) | |

[a]Row percentages. [b]By $\chi^2$ analysis. [c]T: tumour size; N: lymph nodes; ER: oestrogen receptors.

### Probes and allelic deletion analysis

All probes used in this study were as follows: D17S5 (Nakamura et al, 1988) and D17S379 (Carrozzo and Ledbetter, 1993) were the two proximal markers; D17S34 (Isomura et al, 1994) and ABR (Heisterkamp et al, 1993) were the more telomeric markers. The physical distance between these loci has been estimated to be 3 Mb (Figure 1). ABR has been mapped 250 Kb proximal to D17S34 (Stack et al, 1995). Probe YNZ22.1 detects the D17S5 VNTR polymorphisms on Southern blots with *PstI* and *Bam*HI digested genomic DNA; the 144D6 probe detects the D17S34 VNTR on *RsaI* digested DNA. The ABR-VNTR1 polymorphism was detected on *BamI* digested DNA. Southern blot and microsatellite analyses were performed as previously described (Stack et al, 1995). In 17 cases in which D17S5 was heterozygous but LOH needed to be confirmed and in a number of uninformative samples, the D17S379 microsatellite marker was used. D17S379 is linked to D17S5 but is located 200 Kbp proximal (R Carrozzo, personal communication). Tumour/normal products were run in adjacent gel tracks and relative allele intensities were visually compared independently by two individuals. A loss of signal relative to one of the alleles indicated a LOH. In most cases, however, the LOH was not complete and an obvious reduction in intensity of one allele in the tumoural DNA relative to the intensity of normal DNA was observed. This condition has been called allele imbalance; however, for clarity in the present report the term loss of heterozygosity will be used to indicate both of these events. Marginal imbalances and unequal copy number due to amplification were excluded from the statistical analyses.

### TP53 mutation analysis

Polymerase chain reaction (PCR) amplification of exons 5, 6, 7 and 8 covering the highly conserved regions of TP53, was performed with specific primers designed from the flanking intronic sequence of each exon. Mutations were detected by single-strand conformation polymorphism (SSCP) analysis as previously reported (Merlo et al, 1994).

### Cell kinetics

S-phase index was measured by an in vitro 5-bromo-2´deoxyuridine (BrdU) uptake analysis, as previously reported (Merlo et al, 1994). Oestrogen receptors (ER) and Ki-67 immunostaining was performed on frozen sections with an anti-Ki-67 (Dako S.p.A., Milano, Italy) anti-ER (Abbott Laboratories, Chicago, IL, USA) antibodies according to manufacturers' instructions. For all immunohistochemical reactions the ABC complex method was used (Vector Laboratories, Burlingame, CA, USA) according with standard procedures.

### Statistical analysis

To group breast tumours into a low and high proliferation rank we used the median values both for BrdU incorporation and Ki-67 staining. These values were calculated on a data set of over 1000 tumours and were 7.5% and 9.5% for BrdU and Ki-67 respectively; ER cut-off value for positivity was set to 10% stained nuclei. In the analysis of relapse-free survival, second primary cancers and deaths due to other causes were not considered events. Differences in subsets in Kaplan–Meier plots and univariate probabilities were evaluated by log-rank test. Multivariate analyses for disease-free survival (DFS) and overall survival (OS) were performed to fit a Cox proportional hazards model (Cox, 1972). A backward stepwise covariate selection was used to identify the most significant prognostic factors, with variables being eliminated by the maximum likelihood estimate.

### RESULTS

### Genetic analysis

DNA samples from primary breast carcinomas of 152 patients with complete follow-up records were tested for genetic alterations on chromosome 17p. Point mutation analysis by SSCP of the TP53 gene was possible in 90 of these tumours. Twenty-five tumours were found to have a mutated TP53 gene (27.8%). To detect allele losses in the 17p13.3–17pter region four polymorphic

 © Cancer Research Campaign 1999

**Table 3** Univariate analysis of prognostic categorized variables in 143 breast cancer patients: disease-free survival (DFS), overall survival (OS)

| | *P*-values[a] | |
|---|---|---|
| **Variable**[b] | **DFS** | **OS** |
| T (≤ 2 cm vs > 2 cm) | 0.007 | 0.002 |
| N (N– vs N+) | 0.001 | 0.003 |
| ER (ER– vs ER+) | 0.4 | 0.1 |
| BrdU (low vs high) | 0.002 | 0.002 |
| Ki-67 (low vs high) | 0.002 | 0.001 |
| Chromosome 17p13.3 (normal vs LOH) | 0.004 | 0.007 |
| P53 mutations (normal vs mutated) | 0.1 | 0.2 |

[a]By Log-rank test. [b]T: tumour size; N: lymph nodes; ER: oestrogen receptors.

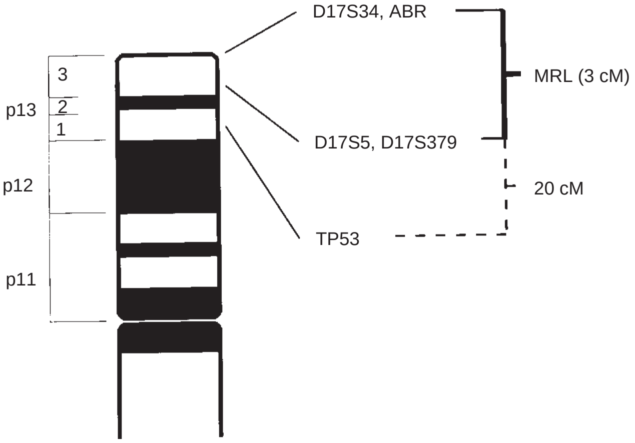

**Figure 1** Idiogram of the short arm of chromosome 17 showing the map order of the markers (not in scale). The minimal region of loss (MRL) and the genetic distances are indicated

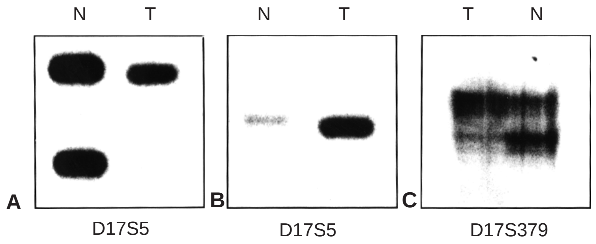

**Figure 2** Autoradiograms from Southern blot analysis demonstrating a loss of heterozygosity (**A**), an uninformative tumour (**B**) that turned out deleted with the microsatellite marker D17S379 (**C**)

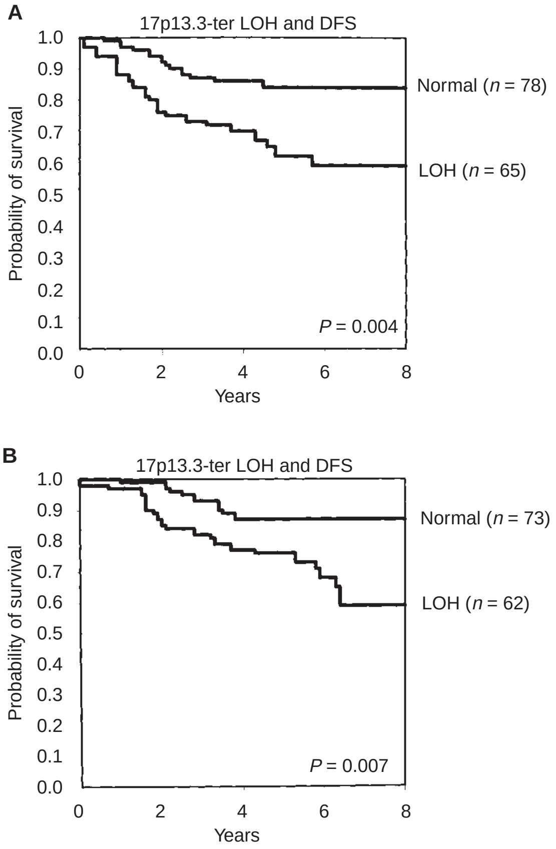

**Figure 3** Kaplan–Meier disease-free (**A**) and overall (**B**) survival curves comparing patients with tumours showing LOH and a heterozygous status of the 17p13.3-ter region. Significance values by log-rank test are indicated loci were examined in the tumour DNA, along with their matching normal lymphocyte DNA. All of the tumours that were found to be deleted at the D17S5 locus showed loss of the D17S379 marker. Due to the lower informativeness compared to D17S5, D17S379 did not provide any additional information on the genomic status of the proximal boundary of the 17p13.3 region. Only in one case was D17S379 found to be deleted while D17S5 was uninformative (Figure 2). Sixty-five of 143 heterozygous tumours had lost at least one of the markers at the minimum region of loss (45%).

### Associations

Our results indicate that the 17p13.3-ter region bordered by loci D17S5/D17S379 and D17S34/ABR is frequently affected by LOH that is independent of point mutation in the P53 gene. To validate the use D17S5/D17S379 and D17S34/ABR for the identification of LOH in a common region of deletion, we tested for the levels of association of losses detected by the centromeric and telomeric markers. The association between D17S5/D17S379 and D17S34/ABR LOHs was highly significant ($P = 0.00001$). In fact, 74.3% of the tumours that had lost the proximal markers had also lost the telomeric polymorphism (Table 1). We were unable to demonstrate any association between LOH of 17p13.3-ter and tumour size, lymph node status, tumour grade, oestrogen receptors, and expression of the cell cycle associated nuclear antigen Ki-67 (Table 2). This later observation is in contrast with the significant relationship observed with the BrdU index ($P = 0.02$), suggesting that chromosome 17p13.3 LOH affects more specifically the S phase of cell cycle. As previously reported (Merlo et al, 1994) a lack of association between LOH of markers on 17p13.3-ter and mutations of the TP53 gene was observed. These data support the belief that in a great proportion of primary breast cancers TP53 is not the actual target of LOH affecting the 17 p subtelomeric region. To ascertain whether adjuvant CT in a set of patients could have influenced the results as a confounding variable, we examined the frequency of deletions in tumours of treated and untreated patients. LOH data were available for 35 tumours of patients who received adjuvant CT. No significant difference in the

© Cancer Research Campaign 1999

**Table 4** Multivariate analysis: proportional hazards regression, Cox model

| DISEASE-FREE SURVIVAL Covariate | RR | 95% Confidence interval | *P*-value |
|---|---|---|---|
| T (≤ 2 cm vs > 2 cm) | 1.1 | (0.3–3.8) | 0.7 |
| N (N– vs N+) | 6.3 | (1.3–29.6) | 0.004 |
| ER (ER– vs ER+) | 1.0 | (0.3–3.5) | 0.5 |
| BrdU (low vs high) | 2.0 | (0.5–8.1) | 0.1 |
| Ki-67 (low vs high) | 1.1 | (0.2–4.3) | 0.2 |
| Chromosome 17p13.3 LOH (normal vs LOH) | 3.1 | (0.9–10.4) | 0.01 |
| TP53 mutation (normal vs mutated) | 1.6 | (0.5–5.1) | 0.2 |
| **OVERALL SURVIVAL** | | | |
| T (≤ 2 cm vs > 2 cm) | 1.3 | (0.3–5.0) | 0.7 |
| N (N– vs N+) | 4.9 | (1.0–24.3) | 0.02 |
| ER (ER– vs ER+) | 0.6 | (0.1–2.6) | 0.4 |
| BrdU (low vs high) | 2.6 | (0.5–12.9) | 0.03 |
| Ki-67 (low vs high) | 1.4 | (0.2–6.9) | 0.6 |
| Chromosome 17p13.3 LOH (normal vs LOH) | 2.5 | (0.6–9.5) | 0.06 |
| TP53 mutation (normal vs mutated) | 1.1 | (0.2–4.2) | 0.6 |

RR = relative risks of relapse or death. T: tumour size, N: lymph nodes, ER: oestrogen receptors.

distribution of allele losses was observed between the two groups (Table 2), so adjuvant CT could not have introduced a significant bias on life-table estimates.

### Survival analysis

LOH of chromosome 17p13.3-ter, lymph node status, tumour size, S phase and Ki-67 all were predictive of disease-specific, relapse-free and overall survival; ER and TP53 gene point mutations were not (Table 3). Analysis of DFS (Figure 3A) showed that patients bearing tumours with a chromosome 17p13.3-ter LOH had a significantly higher probability of relapse than those with tumours that had retained the same chromosome region ($P = 0.004$). By the end of the study after a median follow-up duration time of 6.1 years (range 0.7–8.6 years) among 78 patients with heterozygous tumours the rate of recurrence was 16.6%, while 36.9% of 65 patients with tumours affected by 17p13.3 LOHs had a relapse. In addition 17p13.3-ter LOH appeared to be a good predictor of OS ($P = 0.007$). Among 73 patients with tumours showing a normal status of chromosome 17p13.3-ter, the death rate was 12.3% while 32.2% of 62 patients with tumours carrying LOHs died within 8 years (Figure 3B). Log-rank analysis on a subset of 82 patients, whose tumours were informative for the 17p telomeric markers and could be tested for TP53 point mutation of exon 5, 6, 7 and 8, have shown that while TP53 status, in our hands, did not emerge as significant prognostic indicator ($P = 0.1$) 17p13.3-ter LOH maintained its association both with DFS ($P = 0.01$) and OS ($P = 0.03$) (data not shown). Genetic data were available for 65 of 68 axillary node-negative patients; the number of events, however, were insufficient for demonstrating any prognostic significance of the 17p13.3-ter LOH ($P = 0.2$).

### Multivariate analysis

Even though the aim of our study was not the identification of a new prognostic factor, we attempted to compare the predictive power of 17p13.3-ter LOH with other clinical and biological factors characterizing breast cancer. By proportional hazards regression analysis, using as covariate six other unbiased explanatory variables known to have prognostic significance, 17p13.3-ter LOH have shown an independent hazard ratio for DFS, with a relative risk (RR) of recurrence below that of lymph node status (Table 4). By stepwise regression analysis, performed with a model that takes in account the same concomitant variables, 17p13.3-ter LOH emerged as a significant prognostic factor for DFS ($P = 0.01$) while TP53 was removed from the procedure. For OS, on the other hand, following stepwise selection 17p13.3-ter LOH did not maintain its significance ($P = 0.06$) although its prognostic power was still higher than the TP53 mutational status (2.51 vs 1.12 RR).

### DISCUSSION

In our study, breast cancer specimens of 152 patients were examined for LOH of the short arm of chromosome 17, at band 13.3-ter. The high frequency (45.5%) of this genetic alteration in breast tumours and its association with cell proliferation suggest that the 17p13.3-ter locus is likely to include specific target genes whose inactivation contributes to tumour development or progression. We and others (Merlo et al, 1994) have provided evidence suggesting that these genes are distinct from TP53 and are located within the distal portion of the 17p chromosome arm. The observed 17p loss does not reflect a general telomeric instability of chromosome arms (White et al, 1996). The frequency of 17p13.3-ter LOH reported here may actually be an underestimate of the prevalence of breast cancer allele losses at this locus. Since in the present study no microdissection was attempted, LOH might have been masked by the presence of normal cells in tumour samples. In addition, D17S5 and D17S34/ABR cover 3 Mb of DNA and do not detect interstitial deletions affecting smaller portions of the chromosome arm bordered by these markers; therefore the true extent of this chromosomal alteration is still unknown. We tried to address the question in a previous report (Stack et al, 1995) using a larger number of microsatellites

 © Cancer Research Campaign 1999

spanning the same chromosome band. By these means we were able to identify a distal region in which the frequency of loss was close to 70%; the data collected, however, are at the present insufficient for accurate survival analysis. Although the correlation of 17p13.3 LOH with prognosis have been seen by others in breast cancer (Nagai et al, 1994) an association of this genetic alteration with OS has never been previously investigated. Our results provide evidence that 17p subtelomeric lesions in breast tumours can predict disease-specific survival and, therefore, are associated with a more malignant phenotype. The lack of effect on outcome of 17p13.3 LOH in node-negative patients needs comment. It might be accounted for in part by the small number of observations available for this subset of patients. As shown in another report (Sato et al, 1990) no association was observed between 17p13.3 LOH and lymph node status in our panel of breast cancers. Tumours of node-negative patients seem not to be genetically different from breast carcinomas of patients with lymph node involvement at the time of diagnosis. This notion is supported by a meta-analysis (Mittra and MacRae, 1991) that demonstrates how detection of axillary node metastasis is merely the consequence of a delayed diagnosis and not an indication of actual biological characteristics of tumour tissue. The hypothesis being tested in our study was that breast carcinomas with allele losses at a specific telomeric region of chromosome 17p13.3 could be associated with a diminished survival and, therefore, a more aggressive tumour phenotype. The present report is consistent with this hypothesis and supports the conclusion that a tumour suppressor gene involved in breast cancer, and distinct from TP53, exists on chromosome 17p13. In fact our data fail to demonstrate any predictive power of TP53 point mutations when they are tested, by multivariate analysis, together with cell kinetics and 17p13.3 LOH. Of course we recognize that, since SSCP is only a screening test, our results might have been influenced by the methodology used for the detection of point mutations. However, several data support the belief that TP53 might not play a critical role in breast cancer. More than 70% of breast tumours have no TP53 mutation and the frequency of LOH at the TP53 locus (17p13.1) is low (23%) (Merlo et al, 1994). The most extensive molecular study on TP53 (Sjögren et al, 1996), based on complementary DNA analysis of more than 300 tumours, have brought arguments in favour of an association between p53 alteration and outcome. Its prognostic power, however, was not tested against other genetic alterations. Moreover, an experimental study has shown that suppression of tumorigenicity in breast cancer cells does not necessarily require the transfer of a wild-type p53 gene (Theile et al, 1995). In contrast the role of 17p13.3-ter LOH in breast cancer is stressed by recent evidence suggesting that these chromosomal alteration can occur even in premalignant lesions such as mammary atypical hyperplasias and ductal carcinomas in situ, in which the rate of allele losses have been reported to be higher than 35% (Radford et al, 1995).

Of course the association of LOH of 17p13.3-ter with malignancy only provides circumstantial evidence but not formal proof of the presence of a tumour suppressor gene. The smallest common region of allelic loss on 17p defined by the markers used in our study harbours a number of recently cloned putative tumour suppressor genes. L132/Rox, identified by marker D17S379, has a helix–loop–helix structure characteristic of many transcription factors and binds DNA by forming heterodimers with Max (Meroni et al, 1997). OVCA1 and OVCA2 have been isolated from a cosmid clone, containing the closely linked marker D17S5, that spans a minimal region of allele loss in ovarian cancer (Schultz et al, 1996). The HIC-1 gene has been cloned from a region flanking the D17S5 marker frequently hypermethylated in multiple common types of human cancers, including breast cancer (Makos et al, 1995). The CRK oncogene was originally identified as a transforming component of the avian sarcoma virus CT10. ABR shows homology to BCR, a gene involved in the Philadelphia chromosome translocation in chronic myeloid leukaemia (Heisterkamp et al, 1993). D17S34 is actually located closer to ABR than to CRK, which is over 400 Kb proximal to this marker (Morris et al, 1995). Loss or inactivation, through somatic mutation, of a gene (or genes) mapping on the distal region of the 17p chromosome appears to be a crucial event in the development and progression of sporadic breast cancer as it is able to affect the malignant behaviour of tumours.

### ACKNOWLEDGEMENTS

The authors give their grateful thanks to Prof. Paolo Calderini for providing clinical information. They would also like to thank Prof. Andrea Ballabio and Dr. Romeo Carrozzo for providing information on ROX prior to publication. This work was supported by the Associazione Italiana Ricerca sul Cancro (AIRC)..

### REFERENCES

Bonadonna G, Valagussa P, Tancini G, Rossi A, Brambilla C, Zambetti M, Bignami P, Di Fronzo G and Silvestrini R (1986) Current status of Milan adjuvant chemotherapy trials for node-positive and node-negative breast cancer. *Natl Cancer Inst Monogr* **1**: 45–49

Carrozzo R and Ledbetter DH (1993) Dinucleotide repeat polymorphism mapping to the critical region for lissencephaly (17p 13.3). *Hum Mol Genet* **2**: 615

Coles C, Thompson AM, Elder PA, Cohen BB, Mackenzie IM, Cranston G, Chetty U, Mackay J, Macdonald M, Nakamura Y, Hoyheim B and Steel CM (1990) Evidence implicating at least two genes on chromosome 17p in breast carcinogenesis. *Lancet* **336**: 761–763

Cox DR (1972) Regression models and life tables. *J R Stat Soc* **34**: 187–220

Gudmundsson J, Johannesdottir G, Bergthorsson JT, Arason A, Ingvarsson S, Egilsson V and Barkardottir RB (1995) Different tumor types from BRCA2 carriers show wild-type chromosome deletions on 13q12–q13. *Cancer Res* **55**: 4830–4832

Heisterkamp N, Kaartinen V, van Soest S, Bokoch GM and Groffen J (1993) Human ABR encodes a protein with GAP-rac activity and homology to the DBL nucleotide exchange factor domain. *J Biol Chem* **268**: 16903–16906

Isomura M, Tanigami A, Saito H, Harada Y, Katagiri T, Inazawa J, Ledbetter DH and Nakamura Y (1994) Detailed analysis of LOH on chromosome band 17p13 in breast carcinoma on the basis of a high-resolution physical map with 29 markers. *Genes Chromosomes Cancer* **9**: 173–179

Knudson AG (1985), Hereditary cancer, oncogenes and antioncogenes. *Cancer Res* **45**: 1437–1443

Lancaster JM, Wooster R, Mangion J, Phelan CM, Cochran C, Gumbs C, Seal S, Barfoot R, Collins N, Bignell G, Patel S, Hamoudi R, Larsson C, Wiseman RW, Berchuck A, lglehart JD, Marks JR, Ashworth A, Stratton MR and Futreal PA (1996) BRCA2 mutations in primary breast and ovarian cancers. *Nat Genet* **13**: 238–240

Lindblom A, Skoog L, Andersen TI, Rotstein S, Nordenskjold M and Larsson C (1993) Four separate regions on chromosome 17 show loss of heterozygosity in familial breast carcinomas. *Hum Genet* **91**: 6–12

Mackay J, Steel CM, Elder PA, Forrest AP and Evans HJ (1988) Allele loss on short arm of chromosome 17 in breast cancers. *Lancet* **ii**: 1384–1385

Makos M, Biel MA, Deiry WE, Nelkin BD, Issa JP, Cavenee WK, Kuerbitz SJ and Baylin SB (1995) p53 activates expression of HIC-1, a new candidate tumour suppressor gene on 17p13.3. *Nature Med* **1**: 570–577

Merlo GR, Venesio T, Bernardi A, Cropp CS, Diella F, Cappa APM, Callahan R, and Liscia DS (1994) Evidence for a second tumour suppressor gene on chromosome 17p linked to high S-phase index in primary human breast carcinomas. *Cancer Genet Cytogenet* **76**: 106–111

Meroni G, Reymond A, Alcalay M, Borsani G, Tanigami A, Tonlorenzi R, Lo Nigro C, Messali S, Zollo M, Ledbetter DH, Brent R, Ballabio A and Carrozzo R

(1997) Rox, a novel bHLHZip protein expressed in quiescent cells that heterodimerizes with Max, binds a non-canonical E Box and acts as a transcriptional repressor. *EMBO J* **16**: 2892–2906

Milner J and Medcalf EA (1991) Cotranslation of activated mutant p53 with wild type drives the wild type p53 protein into the mutant conformation. *Cell* **65**: 765–774

Mittra I and MacRae KD (1991) A meta-analysis of reported correlations between prognostic factors in breast cancer: does axillary lymph node metastasis represent biology or chronology? *Eur J Cancer* **27**: 1574–1583

Morris C, Benjes S, Haataja L, Ledbetter DH, Heisterkamp N and Groffen J (1995) Spatial organization of ABR and CRK genes on human chromosome band 17p13.3. *Oncogene* **10**: 1009–1011

Nagai MA, Pacheco MM, Brentani MM, Marques LA, Brentani RR, Ponder BA and Mulligan LM (1994) Allelic loss on distal chromosomes 17p is associated with poor prognosis in a group of Brazilian breast-cancer patients. *Br J Cancer* **69**: 754–758

Nakamura Y, Ballard L, Leppert M, O'Connel P, Lathrop GM, Lalouel JM and White R (1988) Isolation and mapping of a polymorphic DNA sequence (pYNZ22) on chromosome 17p [D17S30]. *Nucleic Acids Res* **16**: 570–

Neuhausen SL and Marshall CJ (1994) Loss of heterozygosity in familial tumors from three BRCA1-linked kindreds. *Cancer Res* **54**: 6069–6072

Radford DM, Fair KL, Phillips NJ, Ritter JH, Steinbrueck T, Holt MS and Donis-Keller H (1995) Allelotyping of ductal carcinoma in situ of the breast: deletion of loci on 8p, 13q, 16q, 17p and 17q. *Cancer Res* **55**: 3399–3405

Sato T, Tanigami A, Yamakawa K, Akiyama F, Kasumi F, Sakamoto G and Nakamura Y (1990) Allelotype of breast cancer: cumulative allele losses promote tumour progression in primary breast cancer. *Cancer Res* **50**: 7184–7189

Schultz DC, Vanderveer L, Berman DB, Hamilton TC, Wong AJ and Godwin AK (1996) Identification of two candidate tumor suppressor genes on chromosome 17p13.3. *Cancer Res* **56**: 1997–2002

Sidransky D, Takashi T, Helzlsouer K, Zehnbauer B, Rausch G, Shelton B, Prestigiacomo L, Vogelstein B and Davidson N (1992) Inherited p53 gene mutations in breast cancer. *Cancer Res* **52**: 2984–2986

Sjögren S, Inganas M, Norberg T, Lindgren A, Nordgren H, Holmberg L and Bergh J (1996) The p53 gene in breast cancer: prognostic value of complementary DNA sequencing versus immunohistochemistry. *J Natl Cancer Inst* **88**: 173–182

Stack M, Jones D, White G, Liscia DS, Venesio T, Casey G, Crichton D, Varley J, Mitchell E, Heighway J and Santibanez-Koref M (1995) Detailed mapping and loss of heterozygosity analysis suggests a suppressor locus involved in sporadic breast cancer within a distal region of chromosome band 17. *Hum Mol Genet* **4**: 2047–2055

Theile M, Hartmann S, Scherthan H, Arnold W, Deppert W, Frege R, Glaab F, Haensch W and Scherneck S (1995) Suppression of tumorigenicity of breast cancer cells by transfer of human chromosome 17 does not require transferred BRCA1 and p53 genes. *Oncogene* **10**: 439–447

White GRM, Stack M, Santibanez-Koref M, Liscia DS, Venesio T, Wang JC, Helms C, Donis-Keller H, Betticher DC, Altermatt HJ, Hoban PR and Heighway J (1996) High levels of loss at the 17p telomere suggest the close proximity of a tumour suppressor. *J Cancer* **74**: 863–870

 *© Cancer Research Campaign 1999*
